# Addressing the Burden of Disease Attributable to Air Pollution in India: The Need to Integrate across Household and Ambient Air Pollution Exposures

**DOI:** 10.1289/ehp.1307822

**Published:** 2014-01-01

**Authors:** Kalpana Balakrishnan, Aaron Cohen, Kirk R. Smith

**Affiliations:** 1 Department of Environmental Health Engineering, Sri Ramachandra University, Chennai, India; 2 Health Effects Institute, Boston, Massachusetts, USA; 3 Division of Environmental Health Sciences, School of Public Health, University of California, Berkeley, California, USA

In the comparative risk assessment (Lim et al. 2012), performed as part of the Global Burden of Disease (GBD) 2010 Project, air pollution ranked as a leading contributor to the burden of disease in South Asia. Estimates of the burden in India show approximately 1.04 million premature deaths and 31.4 million disability-adjusted life years (DALYs) to be attributable to household air pollution (HAP) resulting from solid cooking fuels, and 627,000 premature deaths and nearly 17.8 million DALYs to be attributable to ambient air pollution (AAP) in the form of fine particulate matter ≤ 2.5 µm in aerodynamic diameter (PM_2.5_). HAP and AAP account for 6% and 3%, respectively, of the total national burden of disease, and together they exceed the burden from any other risk factor of the > 60 examined. This burden, borne disproportionately by poor populations who rely on solid fuels for cooking, poses an enormous challenge for air quality management within public health programs in India. There is a need to integrate research and intervention across HAP and AAP exposures in India in order to reduce disease burdens and to efficiently improve health by using intervention efforts.

The HAP exposure model used in GBD 2010 (based on measurements and modeling results from India) estimated daily average PM_2.5_ exposures of 285 µg/m^3^, 337 µg/m^3^, and 204 µg/m^3^ for children, women, and men, respectively (Balakrishnan et al. 2013). The global model used for AAP exposures (which for the first time included ambient air quality of rural areas) estimated a 2010 population-weighted annual mean PM_2.5_ of 27.2 µg/m^3^ in India, up 6% from 1990, with a distribution that included much higher levels in urban and some rural areas (Brauer et al. 2012). These estimates, which significantly exceed the World Health Organization (WHO) Air Quality Guideline (AQG) levels (WHO 2006), underscore the interrelated contribution of these HAP and AAP exposures to the burden of disease in India.

In GBD 2010, these quantitative exposure estimates were coupled with an integrated exposure–response function to estimate the burden of disease from ischemic heart disease, stroke, acute lower respiratory infection, and lung cancer for both AAP and HAP by contrasting risk under current exposure conditions with the theoretical-minimum-risk exposure distribution that would apply if exposure were reduced to an annual mean PM_2.5_ of approximately 7 µg/m^3^ (Lim et al. 2012). The use of the integrated exposure–response function served to fill—by interpolation—the gap in research on HAP and cardiovascular mortality (Pope et al. 2009; Smith and Peel 2010) and allowed more quantitative comparisons between both. As a result, the total attributable disease burden estimates for AAP and HAP in India are considerably higher than previous estimates (WHO 2004). Further, given the high background rates of ischemic heart disease and stroke (Gupta et al. 2008; Murray and Lopez 2013), chronic/noncommunicable diseases are now estimated to account for most of the attributable burden for both HAP and AAP in India, and in the rest of the world (Institute for Health Metrics and Evaluation 2013).

Poor air quality in Indian cities continues to present an ominous picture for health burdens attributable to AAP. Analysis of routinely collected ambient air quality data [Central Pollution Control Board (CPCB) 2012] indicates that annual average concentrations of PM_10_ (≤ 10 µm in aerodynamic diameter) are critically high (defined as > 90 µg/m^3^ by the CPCB) at more than half of the 503 locations monitored across India. The newly revised Indian national ambient air quality standards (NAAQS) for annual average PM_10_ and PM_2.5_ (60 and 40 µg/m^3^, respectively) (CPCB 2012) are comparable to the WHO interim target-1 guideline values, but much higher than the recommended WHO AQG values themselves of 20 and 10 µg/m^3^ (WHO 2006) or the U.S. Environmental Protection Agency (EPA) annual PM_2.5_ standard of 12 µg/m^3^ (U.S. EPA 2012). Importantly, the NAAQS are above the counterfactual annual mean PM_2.5_ (7 µg/m^3^) used in GBD 2010, which means that there would be substantial health burden remaining even if the standards were met. Current projections for transportation (focused on increasing vehicular fleets) and power generation (focused on increasing reliance on coal-based plants) thus need to be examined carefully for their implications for additional insults to air quality (Health Effects Institute 2010; International Council on Clean Transportation 2012; International Energy Agency 2011).

At present, the Indian NAAQS remain focused on cities, with extremely limited rural monitoring in place, even though two-thirds of people in India live in rural areas. With about one-fourth of primary outdoor PM_2.5_ in India attributed to solid household cooking fuels, it will be difficult, and in some areas impossible, to meet current NAAQS without reducing household emissions, in addition to addressing vehicular, industrial, and other emissions from known urban sources (CPCB 2011).

Previous efforts to improve conditions for households using solid cooking fuels in India have been mostly directed at developing fuel-efficient biomass cookstoves, but there have been no explicit health- or AQG-driven benchmarks. There is limited evidence of gainful reductions in emissions and exposure from currently available biomass cookstove technologies (Anenberg et al. 2013); thus, HAP interventions need to include innovative ways to increase access to gas and electricity (the only cooking technologies known to currently meet the theoretical-minimum-risk exposure distribution for HAP and AAP), while simultaneously increasing the impetus for research and development to develop truly clean—not just “improved”—biomass stoves. The WHO is currently developing indoor AQGs to provide guidance on indoor emissions rates necessary for cookstoves to satisfy the pollutant-specific WHO AQGs (WHO 2011). Integration of these WHO indoor AQGs within planned programmatic intervention efforts, such as the National Biomass Cookstoves Initiative of the Ministry of New and Renewable Energy, Government of India, would afford an unparalleled opportunity to derive co-benefits for indoor/outdoor air quality and health for a large, highly exposed population.

Given the ubiquity of sources, the interrelated nature of ambient and household exposures, and the likely commonality of health effects associated with particulate matter pollution, health effects studies that perform integrated analyses across HAP and AAP exposure settings are needed to inform policy actions in India. Such studies should focus on the major adverse effects that underlie the current burden estimates, most importantly cardiovascular disease. There are currently few epidemiologic studies of AAP risks in India or of HAP risks anywhere in the world that focus on how joint exposure to AAP and HAP may interact to produce long-term adverse health effects. Global research partnerships would provide an opportunity to strengthen the evidence for exposure response for multiple chronic disease end points that are now becoming the focus for global disease burdens. This new evidence would allow the design of strategies that bring HAP and AAP jointly under the domain of air quality regulation and chronic disease management in India and elsewhere in the world where such exposures coexist.
